# SARS-CoV-2 vaccination in the first year after hematopoietic cell transplant or chimeric antigen receptor T cell therapy: A prospective, multicenter, observational study (BMT CTN 2101)

**DOI:** 10.1101/2024.01.24.24301058

**Published:** 2024-01-25

**Authors:** Joshua A. Hill, Michael J. Martens, Jo-Anne H. Young, Kavita Bhavsar, Jianqun Kou, Min Chen, Lik Wee Lee, Aliyah Baluch, Madhav V. Dhodapkar, Ryotaro Nakamura, Kristin Peyton, Dianna S. Howard, Uroosa Ibrahim, Zainab Shahid, Paul Armistead, Peter Westervelt, John McCarty, Joseph McGuirk, Mehdi Hamadani, Susan DeWolf, Kinga Hosszu, Elad Sharon, Ashley Spahn, Amir A. Toor, Stephanie Waldvogel, Lee M. Greenberger, Jeffery J. Auletta, Mary M. Horowitz, Marcie L. Riches, Miguel-Angel Perales

**Affiliations:** Vaccine and Infectious Disease, Fred Hutchinson Cancer Center, and Department of Medicine, University of Washington, Seattle, WA, USA; Center for International Blood and Marrow Transplantation Research, Medical College of Wisconsin, Milwaukee, WI, USA; Division of Biostatistics, Medical College of Wisconsin, Milwaukee, WI, USA; University of Minnesota, Minneapolis, MN, USA; Center for International Blood and Marrow Transplantation Research, Medical College of Wisconsin, Milwaukee, WI, USA; Center for International Blood and Marrow Transplantation Research, Medical College of Wisconsin, Milwaukee, WI, USA; Center for International Blood and Marrow Transplantation Research, Medical College of Wisconsin, Milwaukee, WI, USA; Adaptive Biotechnologies Corp, Seattle, WA, USA; H. Lee Moffitt Cancer Center and Research Institute, Tampa, FL, USA; Emory University – School of Medicine, Atlanta, GA, USA; City of Hope, Duarte, CA, USA; The Emmes Company, Rockville, MD, USA; Wake Forest Baptist, Winston-Salem, NC, USA; Mount Sinai Hospital, New York, NY, USA; Memorial Sloan Kettering Cancer Center, New York, NY, USA; University of North Carolina Medical Center, Chapel Hill, NC, USA; Barnes-Jewish Hospital, Washington University, St. Louis, MO, USA; Virginia Commonwealth University, Richmond, VA, USA; University of Kansas, Lawrence, KS, USA; Medical College of Wisconsin, Milwaukee, WI, USA; Memorial Sloan Kettering Cancer Center, New York, NY, USA; Memorial Sloan Kettering Cancer Center, New York, NY, USA; National Cancer Institute, Bethesda, MD, USA; National Marrow Donor Program/Center for International Blood and Marrow Transplant Research, Minneapolis, MN, USA; Lehigh Valley Health Network, Allentown, PA, USA; National Marrow Donor Program/Center for International Blood and Marrow Transplant Research, Minneapolis, MN, USA; The Leukemia and Lymphoma Society, Rye Brook, New York, NY, USA; National Marrow Donor Program/Center for International Blood and Marrow Transplant Research, Minneapolis, MN, USA; Nationwide Children’s Hospital, Columbus, OH, USA; Center for International Blood and Marrow Transplantation Research, Medical College of Wisconsin, Milwaukee, WI, USA; Center for International Blood and Marrow Transplantation Research, Medical College of Wisconsin, Milwaukee, WI, USA; Memorial Sloan Kettering Cancer Center, New York, NY, USA; Weill Cornell Medical College, New York, NY, USA

**Keywords:** SARS-CoV-2, Covid-19, vaccine, transplant, hematopoietic cell transplant

## Abstract

**Background:**

The optimal timing of vaccination with SARS-CoV-2 vaccines after cellular therapy is incompletely understood.

**Objective:**

To describe humoral and cellular responses after SARS-CoV-2 vaccination initiated <4 months versus 4–12 months after cellular therapy.

**Design:**

Multicenter prospective observational study.

**Setting:**

34 centers in the United States.

**Participants:**

466 allogeneic hematopoietic cell transplant (HCT; n=231), autologous HCT (n=170), or chimeric antigen receptor T cell (CAR-T cell) therapy (n=65) recipients enrolled between April 2021 and June 2022.

**Interventions:**

SARS-CoV-2 vaccination as part of routine care.

**Measurements:**

We obtained blood prior to and after vaccinations at up to five time points and tested for SARS-CoV-2 spike (anti-S) IgG in all participants and neutralizing antibodies for Wuhan D614G, Delta B.1.617.2, and Omicron B.1.1.529 strains, as well as SARS-CoV-2-specific T cell receptors (TCRs), in a subgroup.

**Results:**

Anti-S IgG and neutralizing antibody responses increased with vaccination in HCT recipients irrespective of vaccine initiation timing but were unchanged in CAR-T cell recipients initiating vaccines within 4 months. Anti-S IgG ^≥^2,500 U/mL was correlated with high neutralizing antibody titers and attained by the last time point in 70%, 69%, and 34% of allogeneic HCT, autologous HCT, and CAR-T cell recipients, respectively. SARS-CoV-2-specific T cell responses were attained in 57%, 83%, and 58%, respectively. Humoral and cellular responses did not significantly differ among participants initiating vaccinations <4 months vs 4–12 months after cellular therapy. Pre-cellular therapy SARS-CoV-2 infection or vaccination were key predictors of post-cellular therapy anti-S IgG levels.

**Limitations:**

The majority of participants were adults and received mRNA vaccines.

**Conclusions:**

These data support starting mRNA SARS-CoV-2 vaccination three to four months after allogeneic HCT, autologous HCT, and CAR-T cell therapy.

**Funding:**

National Marrow Donor Program, Leukemia and Lymphoma Society, Multiple Myeloma Research Foundation, Novartis, LabCorp, American Society for Transplantation and Cellular Therapy, Adaptive Biotechnologies, and the National Institutes of Health

## INTRODUCTION

Recipients of cellular therapies, including hematopoietic cell transplant (HCT) and chimeric antigen receptor T cells (CAR-T cells), have a high risk for morbidity from infection with severe acute respiratory syndrome coronavirus 2 (SARS-CoV-2), the cause of coronavirus disease 2019 (Covid-19) (1–4). Without pre-existing immunity from previous infection or vaccination, up to 30% of cellular therapy recipients die within 4–6 weeks after infection with SARS-CoV-2 (5,6).

Initial studies of SARS-CoV-2 vaccination demonstrated low immunogenicity in patients with hematologic malignancies receiving chemotherapy and cellular therapies (7–12). Most studies focused on humoral immunity using binding antibody measurements, but evaluation of neutralizing antibodies and T cell responses provide additional data relevant to protection from severe infection (13,14). Studies in cellular therapy recipients have few participants vaccinated within the first 3–12 months, precluding conclusions about the optimal timing of vaccination after cellular therapy (15–28).

Although immune responses to routine vaccines following HCT are known to be diminished, guidelines recommend inactivated vaccines such as influenza as soon as three months after HCT and other inactivated vaccines as early as six months post-HCT (29–33). Therefore, guidelines recommended SARS-CoV-2 re-vaccination as early as three months post-cellular therapy in the absence of real-world data (34,35). While knowledge is rapidly accumulating (36–39), the optimal timing, schedule of vaccination, and immunological correlates for protective immunity after cellular therapy are unknown. To address these knowledge gaps, the Center for International Blood and Marrow Transplant Research (CIBMTR) and Blood and Marrow Transplant Clinical Trials Network (BMT CTN) conducted a multi-center, prospective, observational study of the safety and immunogenicity of SARS-CoV-2 vaccination within 12 months after autologous HCT, allogeneic HCT, and CAR-T cell therapy. We previously reported the findings among the first 175 allogeneic HCT recipients (40). In this report, we present the final analyses from the complete cohort of allogeneic HCT recipients in addition to autologous HCT and CAR-T cell recipients.

## METHODS

### Participants and study design

We prospectively enrolled patients of any age who underwent an allogeneic HCT, autologous HCT, or CAR-T cell therapy and were planning to receive a first post-cellular therapy SARS-CoV-2 vaccine within 12 months of treatment. Type of SARS-CoV-2 vaccine, number of doses, and timing post-HCT were at the discretion of institutional standards or provider preference. The study (CIBMTR SC21–07/BMT CTN 2101) was approved by the Institutional Review Board of the National Marrow Donor Program and opened to enrollment in April 2021. Participants or their legal guardians provided written informed consent. This study follows the Strengthening the Reporting of Observational Studies in Epidemiology (STROBE) reporting guideline for observational studies.

### Procedures

Blood was collected within pre-specified windows of two weeks prior to first vaccination (pre-V1), at least 3 weeks after first vaccination and within one week prior to second vaccination (post-V1), one to five weeks after second and third vaccination (post-V2 and post-V3, respectively), and seven to nine months after enrollment (end-of-study) (Figure S1). Data collection is detailed in the Supplement.

### Testing

#### Binding and neutralizing antibodies.

We tested serum for semiquantitative total IgG to the SARS-CoV-2 spike protein (S) receptor-binding domain with the Roche Elecsys Anti-SARS-CoV-2 S assay (anti-S IgG), qualitative detection of high-affinity antibodies to SARS-CoV-2 nucleocapsid (N) protein (anti-N IgG), and neutralizing antibodies for Wuhan D614G, Delta B.1.617.2, and Omicron B.1.1.529 strains (LabCorp; Burlington, NC; Supplement) (41). The upper limit of quantitation for anti-S IgG was 2,500 U/mL. All samples were tested for anti-S IgG; only baseline samples were tested for anti-N IgG. Neutralizing antibodies were tested at the pre-V1, post-V2, and post-V3 or end-of-study time points in up to 30 chronologically enrolled participants per cellular therapy cohort and vaccine initiation timing subgroup.

#### SARS-CoV-2-specific T cells.

We performed T cell receptor (TCR) variable beta chain immunosequencing of genomic DNA from peripheral blood mononuclear cells (PBMCs) using the ImmunoSEQ Assay (Adaptive Biotechnologies, Seattle, WA) to quantify the absolute abundance of unique SARS-CoV-2–specific TCRs as previously described (42–44). We quantified SARS-CoV-2 TCR breadth, defined as the proportion of total unique TCRs associated with SARS-CoV-2, and depth, defined as the extent to which SARS-CoV-2-associated TCRs expand. Samples were classified as positive, negative, or “no call” (representing samples with insufficient TCR rearrangements) using the T-Detect classifier (Adaptive Biotechnologies, Seattle, WA) based on breadth and depth compared to a reference population of individuals with prior SARS-CoV-2 infection. Testing was performed in the same subgroup of individuals tested for neutralizing antibodies.

#### Multiparametric Flow Cytometric Analysis.

Cryopreserved PBMCs from pre-V1 samples were tested by flow cytometry to quantitate CD19+ B cells and CD4+ T cells (Supplement).

### Statistical analysis

The primary objective was to compare the immunogenicity of SARS-CoV-2 vaccines in patients starting <4 months versus 4–12 months after cellular therapy. Power calculations based on anticipated and actual sample sizes are in the Supplement and Tables S1–S2.

To determine relevant anti-S IgG thresholds for immunogenicity, receiver operating characteristic (ROC) curves were employed using anti-S IgG as a continuous marker and dichotomous outcomes of neutralizing antibodies at the median level (≥5,274 ID50) achieved in a non-immunocompromised cohort vaccinated with two doses of mRNA-1273 (Moderna) in a clinical trial using the same assay (41). The proportions of participants with anti-S IgG levels above this threshold (defined as a positive response) are described with Wald 99% confidence intervals (CIs); response rates were compared using a two-sided Z test of the difference in proportions between timing strata. To adjust for imbalances in baseline variables between timing cohorts, propensity scores for the likelihood of being in the <4-month cohort were constructed using logistic regression. A propensity-adjusted analysis compared positive responses at the post-V2, post-V3, and end-of-study time points between the <4-month and 4–12-month subgroups within each cellular therapy cohort. For each timing subgroup, inverse probability weights were constructed from the reciprocal of the propensity of being included in the cohort; weighted response proportions and their standard errors were computed and used to obtain point estimates and 99% CIs for the propensity-adjusted difference in response rates. We used adjusted logistic and linear regression models to evaluate the impact of vaccination timing and cellular therapy type on anti-S IgG and T cell responses. Results from samples obtained within six months of administration of the monoclonal antibody tixagevimab-cilgavimab (Evusheld, AstraZeneca) were excluded from antibody analyses. P-values <0.01 were used to determine statistical significance to account for multiple comparisons. Analyses were performed using SAS Version 9.4 and R version 4.2.

### Role of the funding source

The funders had no role in study design, data collection, data analysis, data interpretation, or writing of the report with the exception of L.M.G. (Leukemia and Lymphoma Society).

## RESULTS

### Participants and treatment characteristics

We enrolled 231 allogeneic HCT, 170 autologous HCT, and 65 CAR-T cell recipients (N=466 in total) who received ≥1 SARS-CoV-2 vaccine at 34 centers in the United States between April 2021 and June 2022. Overall, 231 (50%) participants were vaccinated <4 months after cellular therapy and 235 (50%) 4–12 months after cellular therapy. Demographic and baseline characteristics are in Table 1 and were similar within each cohort stratified by vaccination <4 months versus 4–12 months after cellular therapy (data not shown). Allogeneic HCT recipients were enrolled earlier in the pandemic than autologous HCT and CAR-T cell recipients. Most allogeneic HCT recipients (77%) were taking immunosuppressive medications at baseline. Characteristics of the subgroup of 151 participants (60 allogeneic HCT, 53 autologous HCT, and 38 CAR-T cell recipients) tested for neutralizing antibodies and SARS-CoV-2-specific T cells were similar to the overall cohort (data not shown).

SARS-CoV-2 vaccination, infection, and treatment characteristics of study participants are in Table 2. The distribution of vaccination timing is depicted in Figure S2. Vaccination for SARS-CoV-2 prior to receipt of cellular therapy was more frequent in the <4-month subgroups, consistent with the observation that these participants tended to be enrolled at a later calendar time when SARS-CoV-2 vaccines were more available. Most participants (n=356, 76%) received ≥3 vaccine doses, and the distribution of the number of vaccine doses was similar in those vaccinated <4 months versus 4–12 months after cellular therapy. At baseline, anti-N IgG was positive in 100 (21%) individuals, least frequent in allogeneic HCT recipients, but similar across vaccine initiation time subgroups. Tixagevimab-cilgavimab and intravenous immunoglobulin (IVIG) were administered prior to one or more samples in 29% and 14% of participants, respectively; both were more common in the CAR-T cell cohort but similar across vaccine initiation time subgroups. SARS-CoV-2 infections were identified in 99 participants after initiating vaccinations and were equally distributed across vaccine timing subgroups but more frequent after CAR-T cell therapy (Table 2).

### Binding and neutralizing antibody responses increased with subsequent vaccination in HCT recipients irrespective of vaccine initiation timing but were unchanged in CAR-T cell recipients initiating vaccines within 4 months

**Figure 1.**
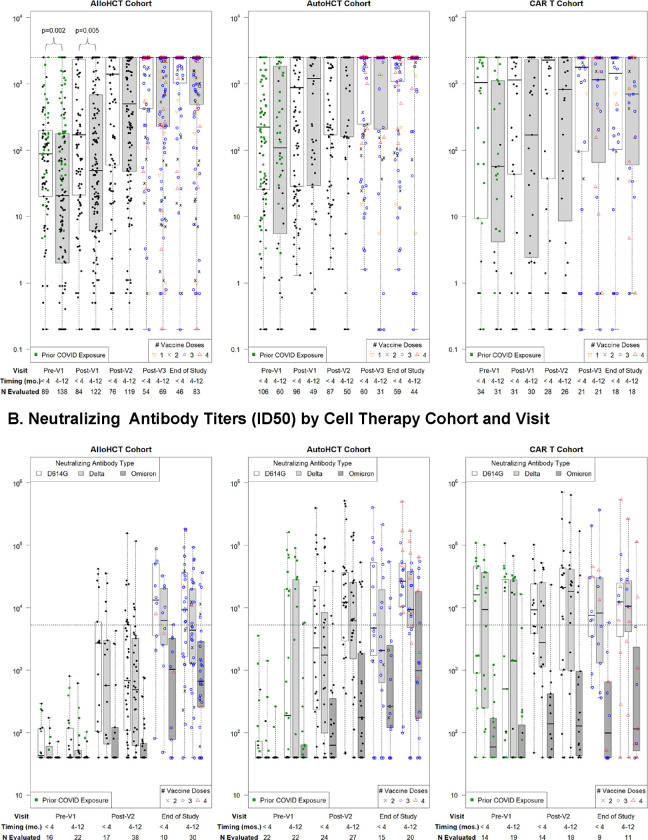
Longitudinal SARS-CoV-2 anti-Spike IgG titers and neutralizing antibody titers stratified by cellular therapy cohort and vaccine initiation timing. **A)** SARS-CoV-2 anti-S IgG titers per time point. The horizontal dotted line indicates the threshold for a positive response, defined as anti-S IgG >2,500 U/mL as determined from a ROC curve analysis; this was also the upper limit of quantitation for the assay. **B)** SARS-CoV-2 neutralizing antibody titers (ID50) in a subgroup of 151 participants; ID50 is defined as the reciprocal of the sample dilution required to reduce relative luminescence units by 50%. The horizontal dotted line shows the median neutralizing antibody level (5,274 ID50) achieved in a healthy cohort vaccinated with two doses of mRNA-1273 (Moderna) in a clinical trial and tested with the same assay and defined here as a positive response. In panels A and B, ‘prior COVID exposure’ (green squares) indicates data in the first two time points from participants with a known prior SARS-CoV-2 infection, prior SARS-Cov-2 vaccination in the participant or hematopoietic cell donor, or positive anti-N IgG assay at baseline. Results are depicted on a log_10_ scale. Time points tested within 6 months of receipt of tixagevimab-cilgavimab (Evusheld) were excluded.

Median anti-S IgG titers were higher in the <4-month subgroups at the baseline (pre-V1) time point in all cohorts but only significantly different in allogeneic HCT recipients (Figure 1A). This difference persisted at the post-V1 time point, but subsequent measurements were similar in all cohorts among those starting vaccines <4 months versus 4–12 months after cellular therapy (Table S3). Anti-S IgG increased at post-V2 and subsequent time points in both vaccine timing subgroups among the allogeneic and autologous HCT cohorts (Table S4). In the CAR-T cell cohort, anti-S IgG titers were relatively unchanged after vaccinations in the <4-month subgroup; in the 4–12-month subgroup, titers increased after two vaccines with a subsequent plateau, but the changes did not reach statistical significance. Results in the CAR-T cell cohort stratified by CD19 versus BCMA-targeted therapies are in Figure S3.

Neutralizing antibody titers increased with vaccinations in the allogeneic and autologous HCT cohorts and were similar at the end-of-study time point by vaccine initiation timing (Figure 1B; Table S3–S4). In the CAR-T cell cohort, similar patterns were seen as for the anti-S IgG results and were comparable at the end-of-study time point by vaccine initiation timing. In all cohorts, most participants had low neutralizing antibody levels for Omicron B.1.1.529.

### The proportion of participants with positive antibody responses increased with vaccination and was similar at the end-of-study time point after HCT, irrespective of vaccine initiation timing; CAR-T cell recipients had declining proportions despite vaccination.

An ROC curve analysis demonstrated that anti-S IgG performed well for predicting high-level neutralizing antibodies with area under the curve (AUC) values of 0.93 and 0.89 in the allogeneic and autologous HCT cohorts, respectively, and 0.72 in the CAR-T cell cohort (Figure S4). Based on these analyses, we defined an anti-S IgG threshold of >2,500 U/mL as a positive response due to correlation with development of high-level neutralizing antibodies across all cohorts.

**Figure 2.**
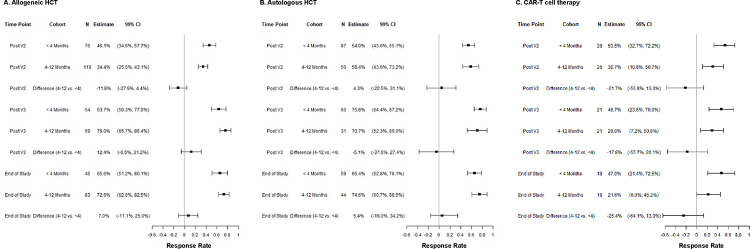
Propensity score-adjusted antibody response rates (anti-Spike IgG >2,500 U/mL) after two or more vaccine doses stratified by cellular therapy cohort and vaccine initiation timing. Forest plots of the proportion of individuals at each time point, stratified by vaccine initiation <4 months versus 4–12 months after cellular therapy, who had a positive anti-Spike IgG value (defined as 2,500 U/mL). The allogeneic HCT, autologous HCT, and CAR-T cell therapy cohorts are depicted in Panels **A, B**, and **C**, respectively. Propensity scores of being in the <4-month timing subgroup were constructed using stepwise variable selection with a criterion of a p-value ≤0.05 to determine which variables were included in the final model. Potential interactions were evaluated between covariates. Cell therapy type, lymphocyte count, and calendar time of enrollment were included in the final model. Inverse probability weights (IPW) were constructed from these propensity scores for all patients according to their timing groups. These were used to obtain adjusted response rate estimates and confidence intervals using IPW proportions and their standard errors. Wald 99% confidence intervals (CI) are shown. Comparisons used a Wald test to compare IPW weighted proportions. Patients who received tixagevimab-cilgavimab (Evusheld) within the prior six months were excluded.

A positive response was attained in 70%, 69%, and 34% of allogeneic HCT, autologous HCT, and CAR-T cell recipients by the last time point in propensity-adjusted analyses that accounted for imbalances in baseline variables between vaccine initiation timing subgroups (Figure 2). Response rates were similar when stratified by vaccine initiation <4 months versus 4–12 months after allogeneic and autologous HCT. In the CAR-T cell group, response rates were twice as high in the <4-month subgroup at the last time point; however, similar differences were seen at the baseline time point, and the proportion of CAR-T cell recipients with anti-S IgG ^≥^2,500 U/mL declined over time in both subgroups despite vaccination.

### SARS-CoV-2-specific T cell responses increased with vaccination in all cohorts and were similar by vaccine initiation timing

**Figure 3.**
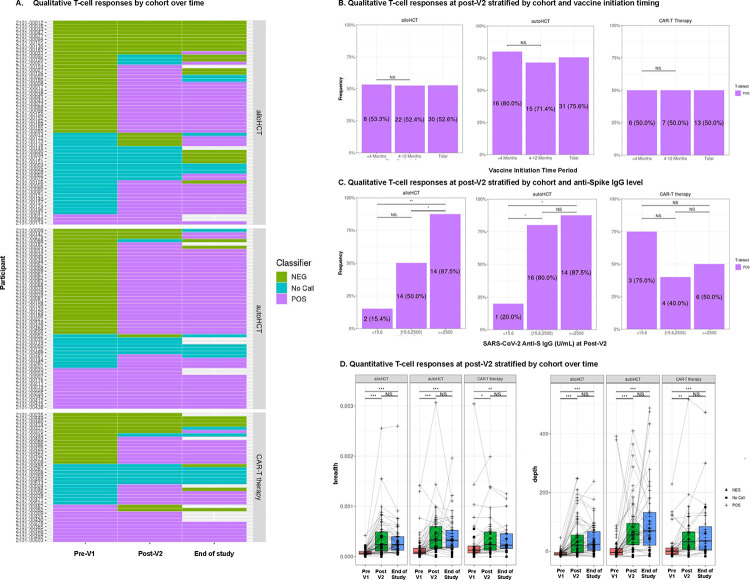
SARS-CoV-2-specific T cell receptor (TCR) variable beta chain sequencing results in a subgroup of 151 participants. **A)** Qualitative results indicating a positive, negative, or indeterminate (‘no call’) result for the presence of SARS-CoV-2-specific TCRs based on the T-Detect ImmunoSEQ Assay classifier (Adaptive Biotechnologies, Seattle, WA). Each row indicates a unique participant clustered by allogeneic HCT recipients at the top of the panel, autologous HCT recipients in the middle, and CAR-T cell therapy recipients at the bottom of the panel. Unfilled cells at the end-of-study time point indicate that no sample was available for testing. **B)** The proportion of participants with a positive T-Detect at the post-V2 timepoint stratified by cellular therapy cohort and vaccine initiation timing subgroup. Participants with a positive test at baseline were excluded from these analyses. **C**) The proportion of participants with a positive T-Detect at the post-V2 timepoint in categories of negative, any detectable, or positive (>2,500 U/mL) SARS-CoV-2 anti-S IgG titers. Any detectable antibody was based on a threshold determined to be predictive of detection of neutralizing antibodies at any level. In panels B and C, individuals with a positive T-Detect at the pre-V1 time point were excluded. (**D)** Quantitative values at each time point indicating the SARS-CoV-2 TCR breadth, defined as the proportion of total unique TCRs associated with SARS-CoV-2, and depth, defined as the extent to which SARS-CoV-2-associated TCRs expand. NS indicates not significant; *, ≤0.05; **, ≤0.01; ***, ≤0.001.

In the subgroup of 151 participants (60 allogeneic HCT, 53 autologous HCT, and 38 CAR-T cell recipients) tested for SARS-CoV-2-specific TCRs, the pre-V1 sample was positive by the T-Detect test assay in three of 60 (5%) allogeneic HCT recipients, 12 of 53 (23%) autologous HCT recipients, and 11 of 38 (29%) CAR-T cell recipients (Figure 3A). This is consistent with the observation that autologous HCT and CAR-T cell recipients were enrolled later compared to allogeneic HCT recipients with more frequent prior vaccination or infection.

At the post-V2 time point, 55%, 81%, and 61% of allogeneic HCT, autologous HCT, and CAR-T cell recipients had a positive T-Detect, respectively, and findings were relatively unchanged at the end-of-study time point (Table S5). Similar proportions of HCT recipients had a positive T-Detect in the <4-month and 4–12-month subgroups; comparisons in the CAR-T cell cohort were limited by sample size (Figure 3B). The proportion of participants with a positive T-Detect increased when compared across categories of no, low, and positive SARS-CoV-2 anti-S IgG levels in the HCT cohorts, but a low proportion (15%−20%) had a positive T-Detect in the absence of antibodies (Figure 3C). Conversely, 75% of CAR-T cell recipients had a positive T-Detect in the absence of SARS-CoV-2 anti-S IgG, and there was no correlation between T cell responses and antibody titers, although observations were limited by sample size.

We next quantified the relative number (breadth) and relative sum frequency (depth) of detectable SARS-CoV-2-specific TCRs. We observed increased depth and breadth at the post-V2 time point in all three cohorts, with stable results at the end-of-study time point (Figure 3D). When stratified by categories of SARS-CoV-2 anti-S IgG titers, breadth and depth increased as the anti-S IgG titer increased in the allogeneic HCT and autologous HCT recipients but not CAR-T cell recipients (Figure S5). Metrics were similar in CD19 versus BCMA-CAR-T cell recipients at the post-V2 time point (Figure S6).

### Pre-cellular therapy SARS-CoV-2 infection or vaccination in the recipient are key predictors of post-cellular therapy SARS-CoV-2 humoral and cellular immunity

We performed multivariable logistic and linear regression models evaluating cellular therapy type, vaccine initiation timing, and other covariates on vaccine response as measured by SARS-CoV-2 anti-S IgG, both as a categorical variable (positive response above versus below 2,500 U/mL) and continuous variable at post-V2, post-V3, and end-of-study time points (Table S6–S7). There were no significant differences in quantitative SARS-CoV-2 anti-S IgG values by vaccine initiation timing subgroup within any of the cellular therapy cohorts. Antibody titers were lower in the CAR-T cell cohort than the HCT cohorts, particularly at the last time point. Throughout most models and time points, prior vaccination or infection with SARS-CoV-2 in the recipient, as well as absolute CD19^+^ B cell counts at enrollment, were the most significant predictors of higher SARS-CoV-2 anti-S IgG values. Similarly, prior SARS-CoV-2 vaccination or infection were associated with higher SARS-CoV-2-specific T cell responses (Table S8).

### Anti-S IgG and T cell responses correlated with subsequent SARS-CoV-2 infection

Comparisons of antibody and T cell measurements at the post-V2 and end-of-study time points with subsequent SARS-CoV-2 infection demonstrated that participants with lower anti-S IgG titers and with a negative T-Detect assay were more likely to be diagnosed with SARS-CoV-2, but the differences did not reach statistical significance (Table S9). Infections were likely underestimated.

### SARS-CoV-2 mRNA vaccination was safe

Possible vaccine-related grade 3 or higher adverse events were uncommon (Table S10). New-onset acute or chronic GVHD were within the expected ranges for this patient population. No CAR-T cell recipients experienced recurrent immune-associated toxicities attributed to vaccination, such as cytokine release syndrome or immune effector cell-associated neurotoxicity syndrome.

## DISCUSSION

In this prospective, multicenter study of 466 allogeneic HCT, autologous HCT, and CAR-T cell recipients who initiated SARS-CoV-2 vaccination with mRNA vaccines within the first 12 months after cellular therapy, we demonstrate that humoral and cellular responses after two or more vaccinations were similar in participants initiating vaccination within <4 months versus 4–12 months. Binding and neutralizing antibody responses increased over time in the HCT cohorts but remained relatively unchanged in the CAR-T cell cohort. However, SARS-CoV-2 specific T cells increased in depth and breadth with vaccination in all cohorts. In adjusted models, pre-cellular therapy SARS-CoV-2 vaccination or infection of the recipient and baseline B cell count were key predictors of post-cellular therapy immunity.

We previously reported results from the first 175 allogeneic HCT recipients enrolled in this study, which demonstrated similar findings of comparable humoral and cellular responses after two or more SARS-CoV-2 mRNA vaccinations initiated <4 months versus 4–12 months after HCT (40). In this manuscript, we include an expanded cohort of allogeneic HCT recipients with increased diversity in race, ethnicity, and age (more pediatric participants) in addition to autologous HCT and CAR-T cell cohorts. The results of our prior study were replicated in the expanded allogeneic HCT cohort and the autologous HCT cohort. Notably, there was no evidence for a humoral immune response in the CAR-T cell cohort initiating mRNA SARS-CoV-2 vaccination <4 months after CAR-T cell infusion. Although binding and neutralizing antibody titers increased overall in the 4–12 month vaccine initiation subgroup, the changes were not significant, and baseline levels started at a lower level than in the <4 month subgroup. In both CAR-T cell subgroups, the proportion of participants with an anti-S IgG titer >2,500 U/mL decreased from the baseline to the end-of-study time point despite vaccination, with only 33% of participants achieving this threshold. It is possible that vaccination maintained antibody titers at a higher level than would otherwise be anticipated after B cell depleting CAR-T cell therapy.

Beyond humoral immune responses, pathogen-specific T cell responses are a critical aspect of assessing immunity to viral pathogens in immunocompromised individuals. T cell responses play a key role in controlling the severity of disease once infected (45,46). An important finding in this study was the development of SARS-CoV-2-specific T cell responses in the majority of participants in all three cellular therapy cohorts, and responses appeared to peak after two vaccine doses, similar to findings in related contexts (7). The proportion of participants with a positive T-Detect assay was similar across vaccine initiation timing subgroups and generally increased when compared across categories of no, low, and positive SARS-CoV-2 anti-S IgG levels in the HCT cohorts, although was not correlated with antibody responses in the CAR-T cell cohort. In a study of T cell responses to SARS-CoV-2 vaccination and infection in individuals lacking B cells due to treatment with rituximab or a primary immunodeficiency, the authors observed greater reactivity and proliferative capacity of SARS-CoV-2-specific T cells elicited by both infection and vaccination in the B cell deficient patients compared to healthy controls (45). Furthermore, they observed a 4.8-fold reduced odds of moderate or severe Covid-19 in vaccinated B cell deficient participants despite the absence of vaccine-induced antibodies. Together, these data underscore the importance of vaccinating CAR-T cell recipients to establish cellular immunity despite an expectation of poor humoral immune responses.

In adjusted models evaluating the impact of cellular therapy type and vaccine initiation timing, we demonstrated that prior vaccination or infection with SARS-CoV-2 in the recipient were the most consistently significant predictors of higher SARS-CoV-2 anti-S IgG and T cell values. The persistence of pre-cellular therapy SARS-CoV-2-specific humoral immunity was apparent based on the frequent presence of antibodies at the baseline time point in all cohorts, with generally higher antibody titers in patients tested earlier after cellular therapy. Notably, we did not observe associations with other clinical variables often considered in immunocompromised patients, such as the presence of GVHD, use of immunosuppressive therapies, or absolute lymphocyte counts (15–18,20–22,24–26).

This study is one of the largest prospective analyses of vaccination within the first year after cellular therapy and supports current guidelines for SARS-CoV-2 vaccination starting approximately three months after cell infusion (35,47). Most guidelines recommend vaccine initiation six to twelve months after cellular therapies, although some advocate initiation as soon as three months (29–31,33,48,49). Our data provide encouraging proof-of-concept for the utility of early vaccination targeting additional pathogens with mRNA vaccine platforms and underscore the importance of investigating multiple aspects of immunogenicity. The observation that neutralizing antibody titers remained low for SARS-CoV-2 variants after Wuhan D614G-targeted vaccination underscores the importance of booster vaccinations, continued utilization and development of prophylactic and therapeutic interventions, and other infection prevention strategies in this population.

A limitation is that this was an observational study, and we may not have fully accounted for confounding, although we used rigorous statistical methodology to account for observed differences. We are unable to account for variables impacting vaccine initiation timing based on center policies or patient-specific considerations. Because vaccine practices evolved over the course of the study, participants recruited earlier in the study were more likely to have initiated vaccination ≥4 months following cellular therapy and less likely to have had prior vaccination or infection. This may explain in part the lower baseline anti-S IgG titers observed in the 4–12-month cohorts; however, we accounted for this variation using propensity-adjusted analyses. We also note that in the allogeneic HCT cohort, data were limited or unavailable for donors in regard to prior SARS-CoV-2 vaccination, prior infection, and SARS-CoV-2 anti-S IgG, which could impact recipient immunity (50,51). Comparisons in the CAR-T cell cohort had lower power due to fewer participants. There was attrition during followup, which could affect our findings. Only two individuals received non-mRNA vaccines, so these results only apply to SARS-CoV-2 mRNA vaccines, and a minority of participants received a fourth vaccine dose. There was limited enrollment of pediatric patients.

In conclusion, these data support ensuring appropriate vaccination of patients prior to receiving cellular therapy in addition to starting mRNA SARS-CoV-2 vaccination within three to four months after allogeneic HCT, autologous HCT, or CAR-T cell therapy.

## Supplementary Material

Supplement 1

## Figures and Tables

**Table 1.** Demographic and clinical characteristics of the participants.

Characteristic	Allogeneic HCT	Autologous HCT	CAR-T	Total
No. of patients	231	170	65	466
Age at cellular therapy, years - no. (%)				
Median (range)	56.5 (3.0–76.7)	60.6 (7.0–77.9)	62.7 (11.0–82.8)	58.6 (3.0–82.8)
<18	20 (9)	3 (2)	2 (3)	25 (5)
18–29	20 (9)	9 (5)	1 (2)	30 (6)
30–39	23 (10)	9 (5)	2 (3)	34 (7)
40–49	26 (11)	18 (11)	7 (11)	51 (11)
50–59	50 (22)	43 (25)	19 (29)	112 (24)
60–69	71 (31)	63 (37)	17 (26)	151 (32)
≥70	21 (9)	25 (15)	17 (26)	63 (14)
Sex - no. (%)			1.00	
Male	120 (52)	97 (57)	35 (54)	252 (54)
Female	111 (48)	73 (43)	30 (46)	214 (46)
Hispanic or Latinx ethnicity- no. (%)				
Yes	14 (6)	13 (8)	5 (8)	32 (7)
No	216 (94)	157 (92)	60 (92)	433 (93)
Unknown/Not reported	1 (0)	0 (0)	0 (0)	1 (0)
Race or Ethnic Group other than Hispanic or Latinx - no. (%)				
White	175 (76)	124 (73)	53 (82)	352 (76)
Black	30 (13)	33 (19)	7 (11)	70 (15)
Asian	15 (6)	6 (4)	4 (6)	25 (5)
American Indian or Alaskan Native	2 (1)	1 (1)	1 (2)	4 (1)
More than one race	3 (1)	1 (1)	0 (0)	4 (1)
Unknown/Not reported	6 (3)	5 (3)	0 (0)	11 (2)
Underlying Disease - no. (%)				
AML/MDS/MPN	147 (64)	0 (0)	0 (0)	147 (32)
ALL/other leukemia/aplastic anemia	59 (26)	0 (0)	5 (8)	64 (14)
Non-Hodgkin lymphoma	14 (6)	32 (19)	37 (57)	81 (17)
Multiple myeloma	0 (0)	117 (69)	23 (35)	140 (30)
Others	11 (5)	21 (12)	0 (0)	32 (7)
Date of cellular therapy - no. (%)				
May, 2020 - December, 2020	22 (10)	6 (4)	2 (3)	30 (6)
January, 2021 - March, 2021	53 (23)	15 (9)	3 (5)	71 (15)
April, 2021 - June, 2021	80 (35)	42 (25)	9 (14)	131 (28)
July, 2021 - September, 2021	56 (24)	35 (21)	11 (17)	102 (22)
October, 2021 - December, 2021	12 (5)	56 (33)	19 (29)	87 (19)
January, 2022 - June, 2022	8 (3)	16 (9)	21 (32)	45 (10)
Number of enrolling centers	30	28	18	34
Graft source - no. (%)				
Bone marrow	37 (16)	1 (1)	0 (0)	38 (8)
Peripheral blood	185 (80)	169 (99)	65 (100)	419 (90)
Cord blood	9 (4)	0 (0)	0 (0)	9 (2)
Donor and HLA match - no. (%)				
Matched related	42 (18)	N/A	N/A	N/A
Matched unrelated	107 (46)	N/A	N/A	N/A
Mismatched related	63 (27)	N/A	N/A	N/A
Mismatched unrelated	19 (8)	N/A	N/A	N/A
Conditioning intensity - no. (%)				
Myeloablative	109 (47)	N/A	N/A	N/A
Reduced intensity	81 (35)	N/A	N/A	N/A
Non-myeloablative or none	41 (18)	N/A	N/A	N/A
GVHD prophylaxis - no. (%)				
Post-HCT cyclophosphamide regimen	90 (39)	N/A	N/A	N/A
Non-post-HCT cyclophosphamide regimen	129 (56)	N/A	N/A	N/A
T cell depletion	12 (5)	N/A	N/A	N/A
Acute GVHD prior to baseline sample - no. (%)				
No	144 (62)	N/A	N/A	N/A
Yes	87 (38)	N/A	N/A	N/A
Chronic GVHD prior to baseline sample - no. (%)				
No	206 (89)	N/A	N/A	N/A
Yes	25 (11)	N/A	N/A	N/A
Immunosuppressive medications in use - no. (%)				
Pre-V1	179 (77)	47 (28)	7 (11)	233 (50)
Post-V2	162 (70)	67 (39)	6 (9)	235 (50)
Post-V3	179 (77)	89 (52)	9 (14)	277 (59)
Post-V4	92 (40)	70 (41)	5 (8)	167 (63)
End-of-study	96 (42)	75 (44)	10 (15)	181 (39)
Absolute lymphocyte count (cells/mm^3^) at baseline, median (IQR)	700 (440–1110)	1100 (700–1640)	605 (320–900)	800 (500–1280)
Absolute CD19+ B cell count (cells/mm^3^) at baseline, median (IQR)	89 (41–183)	139 (46–276)	12 (3–50)	84 (34–198)
Absolute CD4+ T cell count (cells/mm^3^) at baseline, median (IQR)	170 (64–404)	514 (245–870)	194 (90–516)	271 (108–624)

Percentages may not total 100 because of rounding. HCT indicates hematopoietic cell transplant; IQR, interquartile range; AML, acute myelogenous leukemia; MDS, myelodysplastic syndrome; MPN, myeloproliferative neoplasm; ALL, acute lymphoblastic leukemia; HLA, human leukocyte antigen; GVHD, graft-versus-host disease.

**Table 2.** SARS-CoV-2 vaccination, infection, and treatment characteristics of the participants.

Characteristic	Allogeneic HCT	Autologous HCT	CAR-T	Total
No. of patients	231	170	65	466
Donor Vaccination Status Pre-donation - no. (%)				
No	34 (15)	N/A	N/A	34 (7)
Yes	15 (6)	N/A	N/A	15 (3)
Unknown/Not reported	182 (79)	170 (100)	65 (100)	417 (89)
SARS-CoV-2 infection - no. (%)				
Prior to baseline	33 (14)	26 (15)	11 (17)	70 (15)
Before cellular therapy	28 (12)	18 (11)	6 (9)	52 (11)
After cellular therapy	5 (2)	5 (3)	5 (8)	15 (3)
Date Unknown	0 (0)	3 (2)	0 (0)	3 (1)
After initiating vaccinations	44 (19)	36 (21)	19 (29)	99 (21)
Post-V1	4 (2)	5 (3)	3 (5)	12 (3)
Post-V2	14 (6)	12 (7)	2 (3)	28 (6)
Post-V3	25 (11)	15 (9)	14 (22)	54 (12)
Post-V4	1 (0)	3 (2)	0 (0)	4 (1)
Date unknown	0 (0)	1 (1)	0 (0)	1 (0)
Participant vaccinated prior to cellular therapy - no. (%)				
No	183 (79)	66 (39)	19 (29)	268 (58)
Yes	48 (21)	104 (61)	46 (71)	198 (42)
BNT16b2	25 (11)	63 (37)	27 (42)	115 (25)
mRNA-1273	18 (8)	32 (19)	17 (26)	67 (14)
Ad26.COV2.S	2 (1)	6 (4)	1 (2)	9 (2)
Unknown/Not reported	3 (1)	3 (2)	1 (2)	7 (2)
Anti-Nucleocapsid IgG - no. (%)				
Negative	183 (79)	123 (72)	45 (69)	351 (75)
Positive	38 (16)	43 (25)	19 (29)	100 (21)
Unknown/Not reported	10 (4)	4 (2)	1 (2)	15 (3)
Received Vaccine dose 1 - no. (%)				
Yes	231 (100)	170 (100)	65 (100)	466 (100)
BNT16b2	175 (76)	124 (73)	53 (82)	352 (76)
mRNA-1273	55 (24)	45 (26)	12 (18)	112 (24)
Ad26.COV2.S	1 (0)	0 (0)	0 (0)	1 (0)
Unknown/Not reported	0 (0)	1 (1)	0 (0)	1 (0)
Time from cellular therapy to vaccine dose 1, month - median (range)	4.3 (2.2–13.4)	3.6 (1.2–12.1)	3.9 (1.9–8.9)	4.0 (1.2–13.4)
Received Vaccine dose 2 - no. (%)				
No	5 (2)	6 (4)	1 (2)	12 (3)
Yes	219 (95)	160 (94)	63 (97)	442 (95)
BNT16b2	165 (71)	119 (70)	53 (82)	337 (72)
mRNA-1273	53 (23)	41 (24)	10 (15)	104 (22)
Ad26.COV2.S	1 (0)	0 (0)	0 (0)	1 (0)
Unknown/Not reported	7 (3)	4 (2)	1 (2)	12 (3)
Time from cellular therapy to vaccine dose 2, month - median (range)	5.2 (2.9–14.9)	4.5 (1.9–12.9)	4.9 (2.6–9.6)	4.9 (1.9–14.9)
Time between vaccine dose 1 and 2, month - median (range)	0.7 (0.5–3.3)	0.8 (0.3–3.6)	0.8 (0.6–5.2)	0.8 (0.3–5.2)
Received Vaccine dose 3 - no. (%)				
No	43 (19)	27 (16)	13 (20)	83 (18)
Yes	171 (74)	136 (80)	49 (75)	356 (76)
BNT16b2	135 (58)	104 (61)	42 (65)	281 (60)
mRNA-1273	36 (16)	31 (18)	7 (11)	74 (16)
Ad26.COV2.S	0 (0)	1 (1)	0 (0)	1 (0)
Unknown/Not reported	17 (7)	7 (4)	3 (5)	27 (6)
Time from cellular therapy to vaccine dose 3, month - median (range)	7.6 (4.9–18.0)	6.5 (2.9–16.2)	6.5 (4.3–15.0)	7.2 (2.9–18.0)
Time between vaccine dose 2 and 3, month - median (range)	1.6 (0.9–11.6)	1.4 (0.6–9.2)	1.2 (0.7–7.9)	1.5 (0.6–11.6)
Received Vaccine dose 4 - no. (%)				
No	178 (77)	120 (71)	49 (75)	347 (74)
Yes	35 (15)	37 (22)	12 (18)	84 (18)
BNT16b2	27 (12)	30 (18)	10 (15)	67 (14)
mRNA-1273	8 (3)	7 (4)	2 (3)	17 (4)
Unknown/Not reported	18 (8)	13 (8)	4 (6)	35 (8)
Time from cellular therapy to vaccine dose 4, month - median (range)	11.5 (6.0–18.5)	9.5 (7.5–17.1)	9.9 (8.0–12.9)	10.9 (6.0–18.5)
Time between vaccine dose 3 and 4, month - median (range)	4.2 (0.8–7.4)	3.7 (2.4–6.9)	3.0 (1.9–4.3)	3.7 (0.8–7.4)
Receipt of tixagevimab-cilgavimab (Evusheld)^a^ - no. (%)				
Any prior to sample collection	62 (27)	46 (27)	29 (45)	137 (29)
Pre-V1	10 (4)	26 (15)	18 (28)	54 (12)
Pre-V2	1 (0)	5 (3)	0 (0)	6 (1)
Pre-V3	8 (4)	2 (2)	2 (3)	12 (3)
Pre-V4	5 (2)	2 (1)	1 (2)	8 (2)
Pre-end-of-study	45 (19)	21 (12)	12 (18)	78 (17)
Unknown/Not reported	120 (52)	74 (44)	10 (15)	204 (44)
Receipt of IVIG^b^ - no. (%)				
Any prior to sample collection	36 (16)	9 (5)	21 (32)	66 (14)
Pre-V1	14 (6)	3 (2)	7 (11)	24 (5)
Pre-V2	9 (4)	1 (1)	12 (18)	22 (5)
Pre-V3	10 (4)	1 (1)	10 (15)	21 (5)
Pre-V4	7 (3)	1 (1)	8 (12)	16 (3)
Pre-end-of-study	13 (6)	5 (3)	10 (15)	28 (6)
Unknown/Not reported	1 (0)	0 (0)	0 (0)	1 (0)

Percentages may not total 100 because of rounding. HCT indicates hematopoietic cell transplant; IVIG, intravenous immunoglobulin.

a Within 6 months prior to sample collection

b Within 1 month prior to sample collection

## Data Availability

These data will be publically available either on the CIBMTR website or in BioLINCC, the CIBMTR and BMT CTN platforms for public sharing of data.
